# Perceived Risk and HPV Vaccination Awareness Among Women in Rural and Underserved Communities in the State of Louisiana

**DOI:** 10.1007/s10900-025-01465-7

**Published:** 2025-04-05

**Authors:** Deborah Gurgel Smith, Emily M. Dantes, Robbie Beyl, Yanna X. A. de Koter, Margaret Bourg, Corey D. Smith, Gelinia Jackson, Justin Brown, Jerry W. McLarty

**Affiliations:** 1 School of Allied Health Professions, Louisiana State University Health Shreveport, Shreveport, LA USA; 2 School of Medicine, Louisiana State University Health Shreveport, Shreveport, LA USA; 3 Pennington Biomedical Research Center, Baton Rouge, LA USA; 4 Department of Pediatrics, College of Medicine, University of Oklahoma Health Sciences, Oklahoma City, OK USA; 5 Nursing School, Louisiana State University Health New Orleans, New Orleans, LA USA; 6 Feist-Weiller Cancer Center, School of Medicine, Louisiana State University Health Shreveport, Shreveport, LA USA

**Keywords:** Human papillomavirus, Rural health, Cervical cancer, HPV vaccine

## Abstract

Despite the availability of effective preventive measures, women in rural and underserved communities of Louisiana face health disparities regarding human papillomavirus infections. This study explores how perceived risk and socioeconomic factors, such as income, influence HPV vaccine awareness and attitudes toward HPV risk. A cross-sectional study was conducted among women in rural and underserved areas of Louisiana from November 2022 to December 2023. Participants were eligible to be included in the study if they were adult females aged 25 to 64 with no history of hysterectomy and no history of cervical cancer. We used convenience sampling through a mobile health unit that travels to rural and underserved areas of north and central Louisiana, offering cervical cancer screening. A total of 141 women participated in the study. Findings revealed significant gaps in HPV awareness and vaccination knowledge. Only 10.6% of participants considered themselves at risk for HPV. Higher HPV knowledge scores were positively associated with perceived HPV risk, increasing by approximately 20% per correct response. Approximately 40% of the participants were unaware of the existence of the HPV vaccine, 96.5% had never received the HPV vaccine, and 91.4% had never been offered it. Only 42% indicated that they would consider vaccination if offered. Addressing health disparities in rural Louisiana requires targeted interventions to improve healthcare access, education, and community engagement. Efforts to enhance education and awareness and foster community engagement should be prioritized.

## Background

Human papillomavirus is the most common sexually transmitted infection in the United States and causes several diseases [1]. HPV consists of over 200 related viruses, of which 40 are transmitted via sexual contact; among these, two HPV types are responsible for specific cancers and genital warts [2]. Approximately 42 million Americans are infected with HPV, and about 13 million individuals in the US, including teens, become infected each year [3]. About 80% of women will have exposure to at least one type of HPV during their lifetime with no symptoms or clearance of the virus [4].

An HPV vaccine, Gardasil 9, has been available in the United States since 2016 and is recommended starting at age 11 or 12 as a primary prevention method for nine HPV types identified as 6,11,16,18,31,3,45,52, and 58 [2]. While vaccination against HPV is very effective in preventing HPV-related cancers, national vaccination coverage for HPV in the US remains low [5]. In 2020, 58.6% of adolescents in the US were up-to-date with their HPV vaccinations, which is lower than in other high-income countries with successful vaccination programs [6, 7]. In addition to the HPV vaccine, it is recommended that women aged 21 to 29 years with average risk receive cytological screening every 3 years, or every 5 years for those aged 30 to 65 years if receiving the human papillomavirus (HPV) testing alone or HPV co-testing with cytological screening (Pap test) [8].

Despite the availability of effective preventative measures, women in rural communities in the United States face health disparities regarding HPV infections. Screening rates in the southern United States have been low, particularly among women who have not undergone screening in the past five years [9]. In the southern United States, rural populations have a higher HPV-related cancer incidence rate than urban areas [10, 11]. Over the past 50 years, the number of new cases and deaths from cervical cancer screening has significantly decreased [12]. However, the percentage of women in the United States who are overdue for cervical cancer screening has been increasing, and barriers to screening are unclear, especially for women in rural areas. A recent study analyzing data from the National Health Interview Survey revealed that the percentage of women in the United States who were overdue for cervical cancer screenings increased from 14% in 2005 to 23% in 2019 [13]. That study found that in 2019, rates of overdue screening were higher among uninsured (42%) and rural women (26%), revealing disparities across sociodemographic groups.

One barrier to screening for women in rural areas is a lack of access to healthcare. All but five of Louisiana’s parishes are considered Primary Care Health Professional Shortage Areas (HPSA), indicating that there are too few primary care providers in nearly every geographic area [14]. The lack of primary care providers and the fact that much of the state is geographically rural contribute to poor physical access to care. While the care available is insufficient, other barriers exist. The decrease in the number of women undergoing cervical cancer screening despite substantial data supporting its efficacy in reducing cervical cancer deaths is incredibly concerning and should be a priority when studying and addressing public health issues in the United States. To rectify these disparities, we must first understand how socioeconomic factors and health education among rural, underserved communities in Louisiana contribute to these issues. Therefore, this study aimed to identify perceived risk and HPV vaccination awareness among women in rural and underserved areas in Louisiana.

## Methods

### Study Design and Setting

A cross-sectional study was conducted among women in rural and underserved areas of Louisiana from November 2022 to December 2023. Ethical approval was obtained from the Louisiana State University Health Science Shreveport Institutional Review Board (IRB) at (Approval No: 00002027).

## Participants and Data Collection

Participants were eligible to be included in the study if they were adult females aged 25 to 64 with no history of hysterectomy and no history of cervical cancer. We used convenience sampling through a mobile health unit that travels to rural and underserved areas of north and central Louisiana, offering cervical cancer screening. A total of 141 women participated in the study. The self-administered questionnaire consisted of seven sections with multiple-choice or single-choice questions regarding sociodemographic factors, health behaviors, and knowledge and beliefs about the HPV virus and vaccine. Before answering the questionnaire, participants were required to sign an informed consent form. Annual income was self-reported and recorded as a categorical variable. The income variable does not represent household income but rather that of individual participants and does not indicate proximity to the federal poverty line. The HPV knowledge, behavior, and belief variables in this study included HPV Knowledge Score, having ever heard of the HPV vaccine, perceived risk of HPV infection, and adherence to HPV screening recommendations. The HPV Knowledge Score is a continuous variable made by the summation of correct responses to 23 questions concerning HPV and the HPV vaccine. Correct answers were worth one point, while incorrect or “I don’t know” responses were worth zero points, allowing participants to score on a scale of 0 to 23. The “Ever heard of HPV Vaccine” and “Perceived Risk of HPV” variables are used as dichotomous variables, with “No” and “I don’t know” responses combined for the latter. A multiple-choice question asking participants for time since their last Pap was converted into a variable indicating adherence to HPV screening recommendations. Confounders included in the analysis were age, race, education, and adherence to screening recommendations.

### Statistical Analysis

After descriptive statistics on the sample were prepared (see Table 1), a logistic regression was used to explore the association between income and having ever heard of the HPV vaccine. Three models were developed to explore this relationship: an unadjusted, partially adjusted, and fully adjusted model (see Table 2). The partially adjusted model controls for adherence to screening recommendations, while the fully adjusted model also controls for age, race, and level of education. The relationship between the perceived risk of HPV and HPV knowledge was also explored using logistic regression. Once again, unadjusted, partially adjusted, and fully adjusted models were developed and compared, with the addition of income as a confounder in the fully adjusted model (see Table 3). Missing data were not included in the analysis. Data cleaning and analysis were conducted using SAS 9.4.

**Table 1:** Baseline characteristics of study participants

Variable		Total (*n* = 141)
Age (mean ± SD)		55.1 ± 9.4
Female (%)		141 (100.0%)
Race (%)		
African American		76 (53.9%)
Non-African American		65 (46.1%)
Employment status (%)		
Working full time		52 (31.1%)
Working part time		20 (14.3%)
Other^1^		68 (48.6%)
Highest education level (%)		
High school diploma or less		86 (61.9%)
More than high school diploma		53 (38.1%)
Annual Income		
$0 to $24,999		88 (67.7%)
$25,000 to $49,999		27 (20.8%)
$50,000 and greater		15 (11.5%)
HPV Knowledge Score (mean ± SD)		7.7 ± 5.6
Ever Heard of HPV Vaccine (%)		
Yes		86 (39.0%)
No		55 (61.0%)
Perceived risk of HPV (%)		
Yes		13 (9.2%)
No/Not sure		128 (90.8%)
Adherence to screening recommendations^2^ (%)		
Yes		85 (65.9%)
No		44 (34.1%)

^1^ Includes Not working due to full-time studies, Looking for work, No longer able to work, No longer wish to work, and Other.

^2^The U.S. Preventive Services Task Force (USPSTF) recommends a Pap test every three years for women aged 21–65 years [1].

**Table 2:** Unadjusted and adjusted odds for an association between “ever heard of HPV vaccine” and annual income

	Unadjusted^2^		Partially adjusted^a^		Adjusted^b^		
	OR	95% CI	OR	95% CI	OR		95% CI
*Annual Income*							
$25,000 to $49,999^1^	5.25	(1.68, 16.43)	4.63	(1.45, 14.81)	3.95		(1.20, 13.07)
$50,000 and greater	5.93	(1.26, 27.84)	5.79	(1.20, 27.93)	4.81		(0.97, 23.92)

^1^Reference group for annual income is $0 to $24,999.

^2^Reference group for ‘Ever heard of HPV vaccine’ is No.

^a^Model adjusted for adherence to screening recommendations.

^b^Model adjusted age, race, education, and adherence to screening recommendations

**Table 3:** Unadjusted and adjusted odds for an association between HPV risk perception and HPV knowledge score

	Unadjusted		Partially adjusted^a^		Adjusted^b^	
	OR	95% CI	OR	95% CI	OR	95% CI
*Perceived risk*						
Yes	1.23	(1.08, 1.40)	1.20	(1.06, 1.37)	1.21	(1.03, 1.42)

^1^Reference group is Unlikely/Neutral.

^a^Model adjusted for adherence to screening recommendations.

^b^Model adjusted for age, race, education, income, and adherence to screening recommendations

## Results

The study’s sample, with an average age of 55.1 years (SD = 9.4), most African American women (53.9%). Almost 70% had up to a high school diploma, 31.1% reported working full-time, and 67.7% had an annual income of $24,999 or less (See Table 1).

In the unadjusted model, those with an annual income of $50,000 or greater were over five times as likely to have heard of the HPV vaccine (odds ratio, 5.93; 95% CI, 1.26–27.84). After adjusting for confounders such as age, race, and education in the partially adjusted model, this association weakened slightly but remained significant (see Table 2). In the fully adjusted model, which also accounted for adherence to cervical cancer screening, those with an annual income between $25,000 and $49,000 were four times as likely to have heard of the HPV vaccine when compared to the lowest income group (odds ratio, 3.95; 95% CI, 1.20–13.07). However, the association between the highest and lowest income groups did not maintain statistical significance (odds ratio, 4.81; 95% CI, 0.97–23.92). These findings highlight the importance of considering sociodemographic factors when assessing health awareness and suggest that income remains a significant, though less evident, predictor of HPV vaccine awareness in this study population.

The model exploring the relationship between HPV knowledge and HPV risk perception indicated a positive association between the perceived risk of HPV and higher HPV Knowledge Scores, with an approximate 20% increased likelihood of HPV risk perception for each HPV question answered correctly in both the unadjusted and adjusted models. This association remained significant after adjusting for adherence to screening recommendations and sociodemographic factors. Those who perceived themselves at risk for HPV were more likely to have higher knowledge about HPV, highlighting the importance of risk perception in influencing health knowledge.

## Discussion

This study highlights gaps in HPV awareness and vaccination knowledge among women in rural and underserved areas of Louisiana. Although HPV is the most common sexually transmitted infection in the United States, older individuals are traditionally not regarded as being at risk for sexually transmitted infections (STIs) [15]. The low perceived risk of HPV among participants in this study is concerning, as it reflects a lack of understanding about their susceptibility to HPV infection and its potential consequences. While conducting the study, participants reported fear of cancer being detected, embarrassment about a sexually transmitted infection (STI) associated with cancer, lack of screening and vaccination offered by clinicians, and lack of medical insurance to follow a regular provider. Only 10.6% of participants considered themselves at risk for HPV despite living in an area with higher-than-average rates of HPV-related cancers. Similar results were found in previous studies reporting that rural women often underestimate their risk for HPV, which may contribute to lower vaccination and screening rates. Risk perception is a key driver of health behaviors, and its absence underscores the need for targeted education that not only informs but also motivates women to take preventive actions. Lower socioeconomic status is also associated with a higher risk of HPV infection [16]. Ethnicity can also be a risk factor for infection with HPV, as Black and Hispanic individuals have a higher risk of HPV [17]. A history of chlamydial infection may also increase the risk of HPV infection [18].

Half of the participants in this study were unaware that HPV could be transmitted sexually, and 49% did not know an HPV vaccine was available [19]. This raises the concern that women in rural communities are not being informed about infections they are at risk for. Our results are consistent with previous research demonstrating health disparities in HPV knowledge and vaccine uptake in rural communities [20]. Studies have shown that women who live in rural areas, particularly in the southern United States, have higher rates of cervical cancer morbidity and mortality than those in urban areas, with African American and Hispanic women disproportionately affected [16, 21]. These disparities are often exacerbated by socioeconomic challenges, such as low income, limited education, and restricted access to healthcare services. The high percentage of African American participants (53.9%) and those with low income (67.7%) in this study further emphasize the intersection of racial and socioeconomic disparities in HPV-related health outcomes [22].

Another important finding of this study was the link between higher income levels and increased awareness of the HPV vaccine. Although the Affordable Care Act has opened avenues for most patients’ medical insurance to cover preventative care, such as HPV vaccination, without any out-of-pocket cost to the patient, prior research has shown that socioeconomic factors still significantly affect health awareness and access to preventive services [23]. Low awareness of the HPV vaccine across different income levels underscores systemic barriers that go beyond mere financial constraints, including inadequate health education programs and the absence of culturally relevant messaging [23]. A suggested approach to improve HPV vaccine adoption is to enhance understanding of HPV and its vaccine [24] Previous studies have shown that awareness of HPV encourages HPV vaccination [25, 26]. One study revealed that parental knowledge of HPV was the strongest predictor of adolescent HPV vaccination in the United States, United Kingdom, and Australia [27]. Furthermore, one study in a rural population in the United States found that many women in the study believed their adolescent daughters’ risk of both HPV and cervical cancer to be more significant than their own; many participants were more willing to pay a fee to get their children vaccinated than for themselves [28]. This has significant implications for future generations, as there may be potential for parents to have their children vaccinated against HPV even if they aren’t vaccinated themselves. Data such as this presents compelling evidence for the potential efficacy of more rigorous vaccination education in schools and communities, as well as for the implementation of adolescent vaccination programs.

The findings of this study have significant implications for public health practice and policy. To address the gaps identified, public health interventions must focus on culturally- and regionally tailored education campaigns that raise awareness about HPV infection and its vaccine. These campaigns should prioritize rural communities and address misconceptions about HPV transmission, risks, and prevention. Community health workers and local healthcare providers can play essential roles in delivering these messages and building trust within underserved populations. When those in rural areas have consistent relationships with healthcare providers, trust can be built between them, giving patients more confidence in their provider’s advice. Building these relationships is crucial to raising HPV and vaccination awareness and increasing vaccination rates. To do this, however, we must first work to increase access to these medically underserved areas.

From a policy perspective, increasing healthcare resources in rural areas is imperative. Mobile health units, such as the one utilized in this study, can serve as effective platforms for screening, education, and vaccination services. These units drastically increase healthcare access for those in rural areas who may not have access to transportation or have other barriers to traveling to a healthcare provider in a more urban area. However, their reach must be expanded, and their efforts must be supplemented by policies that reduce financial and logistical barriers to healthcare access. Integrating HPV education and vaccination programs into community health initiatives, such as school health programs and local health fairs, can also enhance their effectiveness.

This study addresses a significant public health issue, providing valuable insights into HPV vaccination awareness and perceived risks among women in rural areas who are more likely to be overdue for Pap smear screenings, highlighting a gap in preventive healthcare. Using a mobile health unit for data collection increased accessibility and ensured that the sample included women who might otherwise face barriers to participation. Additionally, the study design allowed for assessing sociodemographic and health behavior factors, contributing to a comprehensive understanding of disparities in HPV-related knowledge and outcomes.

### Study Limitations

The small sample size in this study was due to the nature of participant recruitment and the study design. We conducted a cross-sectional study over one year, recruiting women attending the mobile health unit serving rural and underserved areas. One of the study’s endpoints was to provide HPV screening and assess HPV infection rates. As the study progressed, we encountered participant repetition. Many of the women who initially participated returned for subsequent screenings, limiting our ability to recruit new participants. This, along with the specific focus on HPV infection assessment, further constrained the eligible population, contributing to the smaller sample size. The cross-sectional nature of the study also precludes the determination of causality between sociodemographic factors and HPV awareness or risk perception. Other limitations include convenience sampling, which may introduce selection bias and limit the generalizability of findings. Finally, reliance on self-reported data could result in recall bias, potentially affecting the validity of certain variables.

## Conclusion

To achieve health equity for women in rural Louisiana and similar underserved areas, targeted interventions that address the specific challenges they face are urgently needed. Efforts to improve access to healthcare services, enhance education and awareness, and foster community engagement should be prioritized. Policymakers, researchers, and healthcare providers must collaborate to develop and implement strategies addressing rurality, socioeconomic disadvantage, and health disparities. These strategies require system changes within healthcare practices that can be easily reproducible to improve HPV screening and vaccination rates. Furthermore, these practices require minimal resources, and most, if not all, can be easily implemented into any practice setting. Future research should identify and address barriers to HPV vaccination and screening, including cultural factors, healthcare provider influence, and systemic challenges. Observational, interventional, and implementation research will be essential in designing and evaluating effective solutions. By addressing these disparities, we can work toward a future where all women, regardless of where they live, have equal access to preventive healthcare and are empowered to protect themselves against HPV and its associated health risks.

## Data Availability

Research data supporting the findings of this study are available upon reasonable request from the corresponding author.
